# Construction of a Novel Gene-Based Model for Survival Prediction of Hepatitis B Virus Carriers With HCC Development

**DOI:** 10.3389/fgene.2021.720888

**Published:** 2021-08-31

**Authors:** Yuan Huang, Wen-Ling Tu, Yan-Qiu Yao, Ye-Ling Cai, Li-Ping Ma

**Affiliations:** ^1^Department of Biochemistry and Molecular Biology, School of Bioscience and Technology, Chengdu Medical College, Chengdu, China; ^2^Department of Genetics, School of Bioscience and Technology, Chengdu Medical College, Chengdu, China; ^3^The Second Affiliated Hospital of Chengdu Medical College, China National Nuclear Corporation 416 Hospital, Chengdu, China

**Keywords:** hepatitis B virus carriers, prognosis, gene signature, overall survival, HBV-related HCC

## Abstract

Despite the effectiveness of hepatitis B virus (HBV) vaccination in reducing the prevalence of chronic HBV infection as well as the incidence of acute hepatitis B, fulminant hepatitis, liver cirrhosis and hepatocellular carcinoma (HCC), there was still a large crowd of chronically infected populations at risk of developing cirrhosis or HCC. In this study, we established a comprehensive prognostic system covering multiple signatures to elevate the predictive accuracy for overall survival (OS) of hepatitis B virus carriers with HCC development. Weighted Gene Co-Expression Network Analysis (WGCNA), Least Absolute Shrinkage and Selection Operator (LASSO), Support Vector Machine Recursive Feature Elimination (SVM-RFE), and multivariate COX analysis, along with a suite of other online analyses were successfully applied to filtrate a three-gene signature model (*TP53*, *CFL1*, and *UBA1*). Afterward, the gene-based risk score was calculated based on the Cox coefficient of the individual gene, and the prognostic power was assessed by time-dependent receiver operating characteristic (tROC) and Kaplan–Meier (KM) survival analysis. Furthermore, the predictive power of the nomogram, integrated with the risk score and clinical parameters (age at diagnosis and TNM stage), was revealed by the calibration plot and tROC curves, which was verified in the validation set. Taken together, our study may be more effective in guiding the clinical decision-making of personalized treatment for HBV carriers.

## Introduction

Hepatocellular carcinoma (HCC), one of the most familiar solid tumors all over the world (Kim et al., 2017), is deemed as the sixth most commonly diagnosed cancer and the third leading cause of cancer motility globally (Eric et al., 2015). The risk factors for HCC are diversiform, such as chronic hepatitis B virus (HBV) or hepatitis C virus (HCV) infection, exposure to aflatoxin, tobacco smoking, alcohol abuse, obesity, and non-alcoholic fatty liver disease (NAFLD) (Kuper et al., 2000; Nair et al., 2002; Chen et al., 2010; Ogawa et al., 2010; Golabi et al., 2019). Previous studies had consistently demonstrated that HBV constituted a dominating cause in terms of environmental etiology for HCC (Yu et al., 2014). Patients with HBV have a poor prognosis by reason of the development of cirrhosis, liver failure or even HCC. According to the statistics of World Health Organization (WHO), there were approximately 350 million HBV carriers, of whom nearly 500,000 carriers passed away every year due to cirrhosis and liver cancer (Kumar et al., 2010; Yopp and Singal, 2014). Despite the effectiveness of HBV vaccination for vaccine recipients in reducing the prevalence of chronic or acute HBV infection as well as the incidence of acute hepatitis B, fulminant hepatitis, liver cirrhosis and HCC (Rafael et al., 2000), a large crowd of chronically infected populations, who were at risk of developing cirrhosis or HCC, still maintained. Therefore, there was an urgent need to identify reliable and efficient prognostic signatures or tools to predict the clinical outcomes for making better decisions regarding observation, surgery, drug therapy and conservative treatments for HBV carriers, which would have a great clinical value in addressing these present challenges.

Currently, biomarkers applied to predict overall survival (OS) had exchanged from clinical characteristics, endogenous substances, pathohistological parameters to particular mutated genes (Rimsza et al., 2008; Katunariæ et al., 2014; Tawinwung et al., 2015). However, a series of distinct restrictions on the application of these biomarkers should be concerned. For instance, the single parameter used to predict OS would lead to a wide variability of results and genetic heterogeneity (Ni and Moser, 2018). Moreover, the number of patients to filtrate precise biomarkers was inadequate in most previous studies, which tended to compromise the prediction accuracy and reliability. Thus, it was a better way to establish a comprehensive prognostic system covering multiple signatures to elevate the predictive accuracy. Such as, the six-gene signature model (*NPEPL1*, *VWF*, *LMO7*, *ALDH2*, *NUAK1*, and *TPT1*) could be a robust tool to predict recurrence-free survival (RFS) and castration resistance in prostate cancer (Jun et al., 2021). Also, a novel gene-based model, with five genes (*PADI1, ATP6V0D2, DPP6, C9orf135*, and *PLG*), had better prediction ability in 1-, 3-, and 5-year OS in clear cell renal cell carcinoma (Zhang et al., 2020). On the basis of the above descriptions, computational techniques could also be applied to a large sum of tumor gene expression profiles to screen hub signatures in HBV-positive carriers, which would rapidly provide a broader scale of robust signatures for their survival prediction.

**FIGURE 1:**
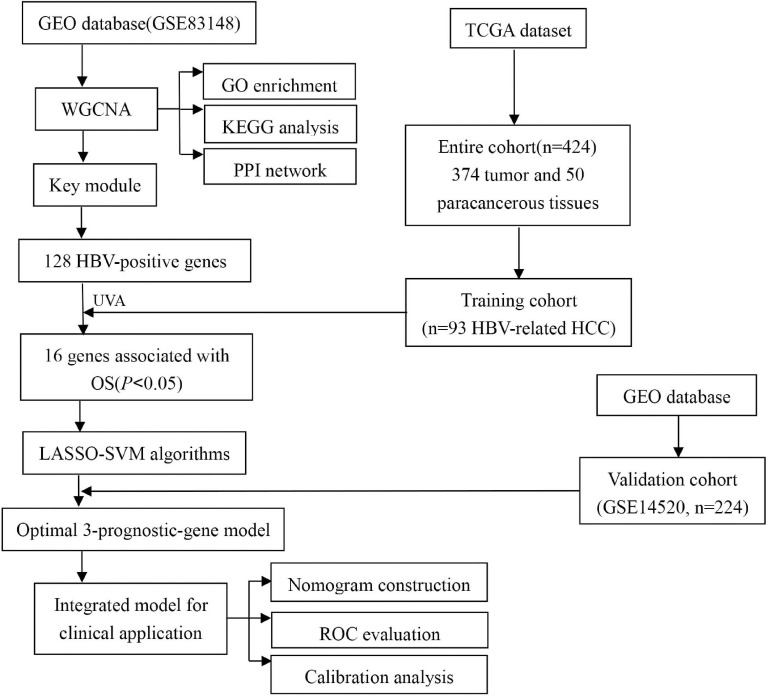
The workflow of our study.

In this study, we constructed a prediction model based on multiple prognostic-related genes as well as clinical parameters to predict OS of HBV-related HCCs. We screened high-throughput sequence dataset and performed a multi-perspectives and multi-dimensional analysis of vast HBV-infected patients with massive bioinformatics and machine learning methods, such as Weighted Gene Co-Expression Network Analysis (WGCNA) (Niemira et al., 2019), Least Absolute Shrinkage and Selection Operator (LASSO) (Vasquez et al., 2016), and Support Vector Machine Recursive Feature Elimination (SVM-RFE) (Sanz et al., 2018). Finally, we identified a three-gene group that may be important prognostic features in predicting OS of HBV-related HCC for HBV carriers. The risk score was calculated through the multivariate cox coefficient multiplied by gene expression. Then the validation cohort was implemented to certify the risk score model. Ultimately, the risk score and clinical parameters were combined together to construct a nomogram, which was assessed by the calibration plot and time-dependent receiver operating characteristic curve (tROC) analysis. The workflow was showed in Figure 1.

## Materials and Methods

### Datasets Sources and Processing

Gene expression profile GSE83148 of 60 HBV-related liver samples was downloaded from GEO database^1^ and used to confirm key module between HBV-positive (*n* = 54) and HBV-negative (*n* = 6) samples with WGCNA after eliminating 68 samples without HBV-DNA or the concentration of HBV-DNA under 1 × 10E6. GSE83148 was performed on GPL570 [HG-U133_Plus_2] Affymetrix Human Genome U133 Plus 2.0 Array. The criteria for selecting the dataset included: (1) All hepatitis samples were HBV infected, which was validated by positive HBsAg and serum HBV-DNA; (2) The samples with HCV infection or metabolic liver injury (e.g., fatty liver, chronic alcoholic hepatitis, etc.) were excluded; (3) HBV-negative samples were collected from 6 healthy people. Besides, dataset GSE114783 performed on NimbleGen Human Gene Expression 12 × 135K Array, consisting of 36 peripheral blood mononuclear cells (PBMCs) samples from 3 healthy people (HP), 3 HBV carriers (HBVC), 10 chronic hepatitis B patients (CHB), 10 liver cirrhosis (LC), and 10 HCC patients, was analyzed to validate the prediction efficiency of hub genes during hepatocarcinogenesis. For model development, RNA-sequencing data and updated clinical data of liver hepatocellular carcinoma (LIHC) cohort from 374 HCC and 50 paracancerous samples were downloaded from the TCGA data portal^2^. According to the virus history, 93 HBV-related HCC patients were screened. Furthermore, one microarray dataset GSE14520 which included 224 HBV-related HCC patients with corresponding clinical information was downloaded for the external validation. It was performed on Affymetrix HT Human Genome U133A Array platform. The clinicopathological information for the enrolled cohorts was summarized in Table 1.

**TABLE 1:** Characteristics of patient cohorts.

**Characteristics**	**Training cohort**	**Validation cohort**
	**TCGA (*n* = 93)**	**GSE14520 (*n* = 224)**
**Gender (%)**		
Female	15 (16.1)	29 (12.9)
Male	78 (83.9)	195 (87.1)
**Age (year) (%)**		
≤65	70 (75.3)	199 (88.2)
>65	23 (24.7)	25 (11.2)
**TNM stage (%)**		
T1 + T2	84 (90.3)	174 (77.7)
T3 + T4	9 (9.7)	50 (22.3)
**Fetoprotein (%)**		
≤300	26 (27.9)	–
>300	67 (72.1)	–
**Survival status (%)**		
Dead	18 (19.4)	86 (38.4)
Alive	75 (80.6)	138 (61.6)

### Selection of HBV-Related Genes

To sift out genes associated with positive HBV, Weighted Gene Co-Expression Network Analysis (WGCNA) was performed with the WGCNA R package (version 3.61^3^), by which highly correlated mRNAs could be classified into the same co-expression modules. There were six steps to screen the hub module with WGCNA (G Pei et al., 2017): (1) calculation of the expression and connectivity correlations of mRNAs between HBV-positive and HBV-negative samples in GSE83148 dataset; (2) election of the soft threshold power (β) in the light of the scale-free topology criterion; (3) estimation of the topological overlap matrix dissimilarity between genes in GSE83148 to establish the dendrogram and distinguish the modules by the dynamic tree cut method; (4) evaluation of the preservation (Z-score > 5 and *p* < 0.05) of modules in HBV-positive and HBV-negative samples with the module preservation statistics; (5) enrichment of differentially expressed genes (DEGs) to modules with the hypergeometric algorithm [f (k, N, M, n) = C (k, M)^∗^C (n-k, N-M)/C (n, N)]; and (6) relation of coexpression modules with positive HBV. According to the results of module-trait relationships, modules showing high correlation were chosen as further research objects, and genes in the key module were denominated as HBV-related genes.

To explore the potential molecular events and pathways involving positive HBV, Gene Ontology (GO) and Kyoto Encyclopedia of Genes and Genomes (KEGG) analyses of HBV-positive genes were carried out through the clusterProfiler R package (version 3.61^4^). GO terms or KEGG pathways with *p* < 0.05 were considered statistically significant. Protein-protein interactions (PPI) of hub genes in the key module were identified by the Search Tool for the Retrieval of Interacting Genes (STRING, version 10.5^5^) (Shannon, 2003). The genes pairs interaction score > 0.4 was selected to construct network. Then the PPIs between hub genes were visualized and analyzed with Cytoscape software (version 3.7.2) (Doncheva et al., 2018).

### Identification of HBV-Associated Genes to Predict Prognosis for HBV Carriers With HCC Development

Univariate Cox regression analysis was performed to assess genes associated with OS in the TCGA training cohort. The HBV-associated genes significantly associated with OS of HBV-positive HCCs were considered as the candidate gene set. Subsequently, Least Absolute Shrinkage and Selection Operator (LASSO), a high dimensional data analysis method, was utilized for biomarker selection in that it could synchronously conduct regularization and variable selection, which would improve the prediction accuracy and effectiveness (Martin-Gomez and Tulla-Puche, 2018). The LASSO algorithm was applied with the glmnet R package (version 3.61^6^). However, the prediction accuracy of most current software was not very high. To solve the problem, this study investigated a software defect prediction model by combining LASSO and Support Vector Machine Recursive Feature Elimination (SVM-RFE) Algorithms together. SVM-RFE, a machine learning method based on support vector machine, is used to find the best variables by deleting SVM-generated eigenvectors (Sahran et al., 2018). SVM module was established to further identify the diagnostic value of biomarkers by e1071 R package (version 3.61^7^). Ultimately, we combined the genes from either LASSO or SVM-RFE algorithm for further analysis. A two-sided *p* value < 0.05 was considered to be statistically significant.

### Validation of Candidate Hub Genes During Hepatocarcinogenesis

Prognostic biomarkers were testified by The Human Protein Atlas^8^ and KMPLOT^9^, which are online expression softwares and survival analysis to assess the protein expression level and prognostic value of genes, separately. Furthermore, with the R package ggpubr (version 3.61^10^), the expression level of three candidate genes in GSE114783, comprising PBMCs samples from 3 HP, 3 HBVC, 10 CHB, 10 LC, and 10 HCC patients, was displayed to verify the specificity and availability of genes during hepatocarcinogenesis.

### Construction and Estimation of a Prognostic Model for HBV-Infected Carriers With HCC Development

The risk score for every patient’s OS was calculated on the foundation of a linear incorporation of the regression coefficient derived from the multivariate Cox regression model and the expression level of the candidate genes (i.e., the signature genes). Thus, the risk score was computed as follow: Risk score = (gene1 expression × gene1 coefficient) + (gene2 expression × gene2 coefficient) + … + (gene N expression × gene N coefficient). A linear combination method was applied to estimate the expression level and coefficient of each gene to get a risk score formula, which was as follows:

$$Riskscore=\sum_{i=1}^{3}\beta i*Expi$$

Exp was the expression level of each candidate gene, and β was the corresponding regression coefficient. On the basis of the median risk score as the cutoff, 93 HBV-positive HCC patients in the training set were classified into high-risk and low-risk groups. Then, according to the Kaplan-Meier survival curve analysis and log-rank test, the OS difference between the high-risk group and the low-risk group was compared. The predictive accuracy of the risk score was computed through the area under the curve (AUC), which was calculated from the tROC curve with the pROC R package (version 3.61^11^). The predictive risk model was then validated in external validation cohort from GEO database.

### Validation of Prognostic Risk Model

For external validation, the external validation set (*n* = 224) was utilized to validate the predictive capability and applicability of the risk model in HCC patients. The risk score of each patient was calculated with the coefficients of the candidate genes. Then the patients were stratified into high-risk and low-risk groups by the median risk score from the training set. The KM survival analysis with log-rank test and tROC analysis were used to validate the prognostic risk model.

### Construction and Estimation of the Nomogram

Firstly, the significance of each variable including clinical features (Gender, Age, Creatinine, Child-Pugh score, TNM stage, Ishak fibrosis score, Fetoprotein, Albumin, Bilirubin, Histologic grade, and Platelet) and risk score in the training cohort was evaluated by multivariate Cox regression analysis for determining the independent risk factors of OS. Afterward, a nomogram was formulated based on the former results of multivariate Cox regression analysis (Roemeling et al., 2007) and by using the rms R package (version 3.61^12^). The predictive performance of the nomogram was measured by Harrell’s concordance index (C-index), calibrated with 1,000 bootstrap samples, and assessed by comparing nomogram-predicted vs. observed Kaplan–Meier estimated survival probability (Du et al., 2018).

### Statistical Analysis

All statistical analyses were conducted with the R software (Windows version 3.6.1). The multivariate Cox proportional hazards regression analyses were performed using the SPSS software (version 17.0; IBM, Chicago, IL, United States). The WGCNA, ClusterProfiles, Forest plot, LASSO, SVM-RFE, tROC curves, nomogram and calibration plots were drawn with the R software. The PPI network was drawn by Cytoscape. All statistical tests were two-sided, and *p* < 0.05 was considered statistically significant.

## Results

### Determination of the Most Relevant HBV-Positive Module by WGCNA

**FIGURE 2:**
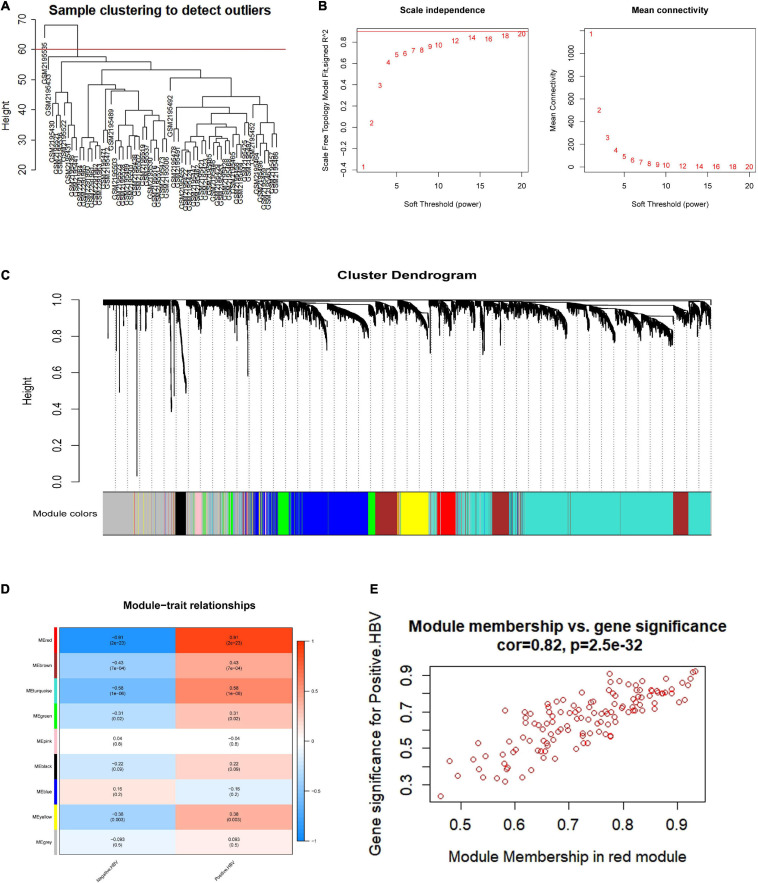
Coexpression network establishment to identify modules related with HBV infection based on WGCNA. **(A)** HBV-related samples clustering to detect outliers. **(B)** The mean connectivity and scale-free fit index with different soft-thresholding powers. **(C)** A clustering dendrogram of coexpression modules screened on the basis of HBV-related samples from GSE83148. **(D)** Module-trait relationships between gene modules and HBV infection. **(E)** The correlation between module membership and gene significance in red module. HBV, hepatitis B virus.

To investigate the hub genes in the HBV-positive samples, WGCNA was applied to one microarray dataset (GSE83148) generated from 54 HBV-infected and 6 non-HBV-infected liver samples. A total of 1,530 genes with | log2 (fold change)| > 1 and *p* < 0.05 were selected for subsequent analysis. By sample clustering analysis to detect outliers, one sample was removed (Figure 2A). The soft threshold power 14 was chosen to create networks with a scale-free topology (R^2^ reached 0.9 for the first time; the mean connectivity was 0) (Figure 2B). Furthermore, the coexpression matrix was constructed and eight gene modules were obtained with the dynamic mixed shear method (Figure 2C). After that, the relevance between eight gene modules and positive HBV was displayed by heatmap, and the correlation between red module and HBV infection was 0.91 (*p* = 2e-23) (Figure 2D). Furthermore, the correlation between gene significance (GS) and module membership (MM) was displayed, indicating that red module (*n* = 128 genes) may be particularly crucial for the infection of HBV (*r* = 0.82, *p* = 2.5e-32) (Figure 2E). Consequently, red module could be regarded as a hub module, which was closely related to HBV infection.

**FIGURE 3:**
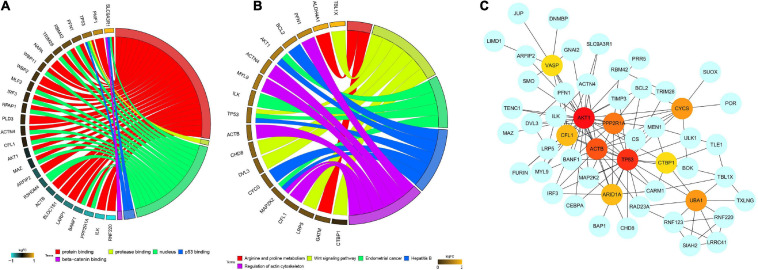
Functional enrichment analysis, including **(A)** GO enrichment analysis and **(B)** KEGG enrichment analysis, and **(C)** PPI network construction of the red module. GO, gene ontology; KEGG, kyoto encyclopedia of genes and genomes; PPI, protein-protein interaction network.

Furthermore, in order to elucidate the possible biological function and the prospective signaling pathways of the hub module, GO annotation and KEGG pathway enrichment analyses were performed on the genes in the red module. Several biological processes were highly enriched in the GO analysis, such as protein binding, protease binding, nucleus, p53 binding, and beta-catenin binding (Figure 3A). With regard to the KEGG analysis, the hub module was focused on arginine and proline metabolism, wnt signaling pathway, endometrial cancer, hepatitis B, and regulation of actin cytoskeleton (Figure 3B). PPI network of red module-associated genes was constructed using the STRING online database and Cytoscape software (Figure 3C). A network containing a total of 128 genes and 159 nodes were identified. Then, the hub genes in the networks with a connectivity degree > 10 were identified. The most significant 10 node degree genes were *TP53*, *AKT1*, *CFL1*, *ACTB*, *PPP2R1A*, *UBA1*, *ARID1A*, *CTBP1*, *CYCS*, and *VASP*, respectively.

### Candidate Hub Gene Selection for HBV-Infected Module

**FIGURE 4:**
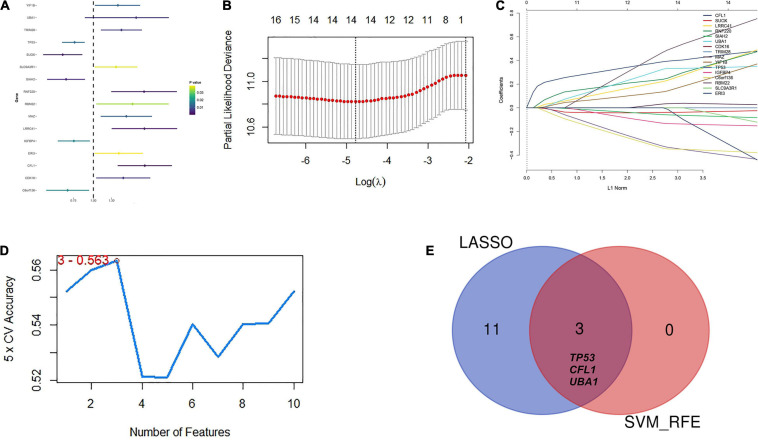
Identification of candidate hub genes to predict prognosis for HBV carriers with HCC development. **(A)** Univariate COX regression of the 128 previously screened variables in the TCGA training cohort (*n* = 93). **(B)** Partial likelihood deviance of different numbers of variables revealed by the LASSO regression model. The red dots represent the partial likelihood deviance values. The gray lines represent the partial likelihood deviance ± standard error (SE). **(C)** LASSO regression with tenfold cross-validation obtained 14 candidate genes with minimum lambda value. **(D)** Candidate genes filtrated from SVM-RFE algorithms. **(E)** A Venn diagram to display the overlapped 3 optimal hub genes between LASSO and SVM-RFE algorithm. LASSO, least absolute shrinkage and selection operator method; SVM-RFE, support vector machine recursive feature elimination.

To confirm the independent prognostic impact of single gene, we performed univariate COX regression of the 128 above screened genes in the TCGA training cohort (*n* = 93), and obtained 16 genes that met the prognostic criteria, identifying a significant association with OS in HBV-related HCCs (*p* < 0.05) (Table 2; Figure 4A). Hence, 16 prognostically relevant signature genes were applied to the LASSO Cox regression model to construct a predictive model for the overall survival of HCC patients with positive HBV in the training cohort (*n* = 93). A 14 genes were selected according to the partial likelihood deviance method, and the corresponding coefficients were generated at the optimal log λ of −4.75. The results are shown in Figures 4B,C. Also, three genes were identified with the SVM algorithm at the best 5 × CV Accuracy (Figure 4D). Furthermore, three shared biomarkers for HBV-related HCC were defined by overlapping the biomarkers derived from these two algorithms (Figure 4E). The three genes (*TP53*, *CFL1*, and *UBA1*), associated with the prognosis of HBV-related HCC, were considered to be the best features.

**TABLE 2:** The univariate COX analysis of the signature.

**Gene**	**HR**	***z***	***p* value**
*CFL1*	2.099549734	3.721961714	0.000197681
*SUOX*	0.643285173	–3.067623172	0.002157685
*LRRC41*	2.088971304	3.058739544	0.002222703
*RNF220*	2.077513627	3.029868836	0.0024466
*SIAH2*	0.675074667	–2.82284238	0.004759997
*UBA1*	1.854675355	2.783812056	0.005372415
*CDK16*	1.531530226	2.709473215	0.006739015
*TRIM28*	1.499066271	2.68776153	0.007193275
*MAZ*	1.60831191	2.522169263	0.011663357
*YIF1B*	1.424721496	2.491148017	0.012733106
*TP53*	0.763297241	–2.465858397	0.013668536
*IGFBP4*	0.755087225	–2.379326413	0.01734431
*C6orf136*	0.690402856	–2.35144127	0.018700842
*RBM22*	1.758736952	2.125391569	0.033553953
*SLC9A3R1*	1.386189279	2.081613	0.037377833
*ERI3*	1.443900641	2.0634463	0.039070245

### Expression Level and Prognosis Analysis of Candidate Hub Genes During Hepatocarcinogenesis

**FIGURE 5:**
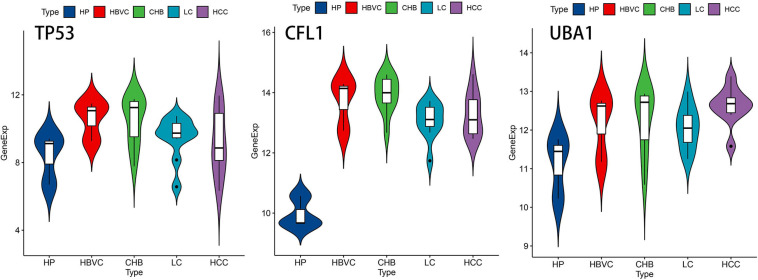
The expression level of *TP53*, *CFL1*, and *UBA1* during multistep hepatocarcinogenesis. Violin plots of the three genes’ expression level during hepatocarcinogenesis on the foundation of dataset GSE114783. HP, healthy people; HBVC, hepatitis B virus carriers; CHB, chronic hepatitis B; LC, liver cirrhosis; HCC, hepatocellular carcinoma.

**FIGURE 6:**
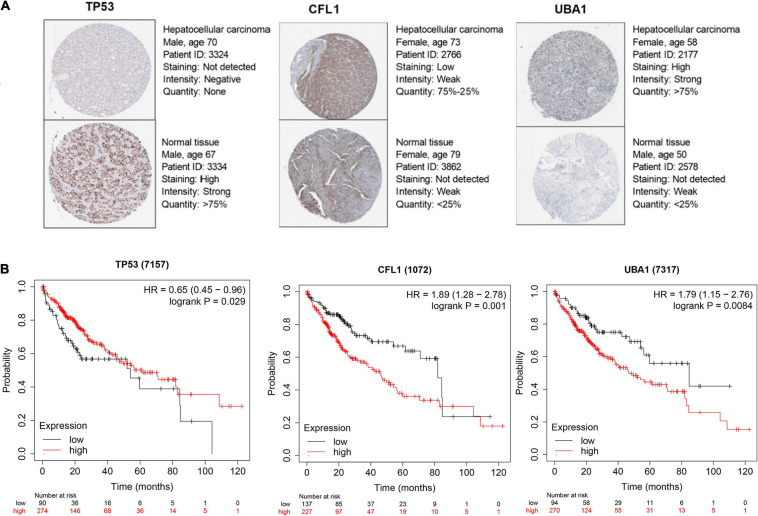
Candidate hub genes selection for prognosis prediction of HCC patients. **(A)** Protein expression level of TP53, CFL1, and UBA1 certified by The Human Protein Atlas database. **(B)** Kaplan–Meier survival analysis of *TP53*, *CFL1*, and *UBA1* from KMPLOT database. KMPLOT, Kaplan-Meier plotter.

In consideration of the multistep hepatocarcinogenesis, three candidate genes’ expression level of PBMCs from healthy people (HP), hepatitis B virus carriers (HBVC), chronic hepatitis B (CHB), liver cirrhosis (LC), to HCC, were explored to investigate the potential specificity and accuracy for predicting OS of HBV-related HCC patients developed from HBV carriers. In PBMCs samples from GSE114783, the expression level of *TP53*, *CFL1*, and *UBA1* was abnormally expressed during HCC development (Figure 5). Moreover, The Human Protein Atlas database was used to validate the protein expression of the three genes. CFL1 and UBA1 were significantly upregulated and TP53 was significantly downregulated in HCC compared with normal liver tissue (Figure 6A). Kaplan-Meier risk curve from KMPLOT database revealed that overexpression of *CFL1* and *UBA1* and low expression of *TP53* were associated with the poor prognosis of HCC patients (Figure 6B). In summary, the expression level of three signatures sustained aberrant during hepatocarcinogenesis.

### Verification of a Prognosis–Related Model for HBV-Positive Carriers

**FIGURE 7:**
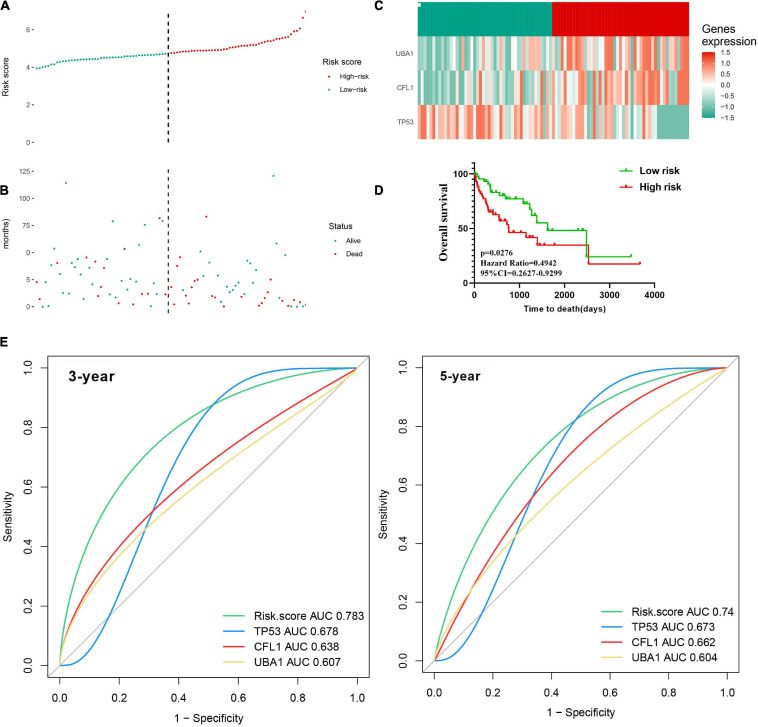
Construction of the three-gene signature in the training cohort to predict OS for HBV carriers with HCC development. The patients were classified into low-risk and high-risk groups by the dotted line, representing the median risk score. Assignment of **(A)** risk score, **(B)** survival status of the patients, and **(C)** the expression profiles of the three risk genes in the training cohort, separately. **(D)** Kaplan–Meier curves of OS according to the risk score in the training cohort. **(E)** Time-dependent ROC curves of the risk score were adopted to estimate the predictability of OS at 3 years and 5 years in the training cohort. OS, overall survival; ROC, receiver operating characteristic.

For each patient, a prognostic risk score was estimated based on the multiplication of a gene’s expression level with its corresponding regression coefficient derived from the multivariate Cox regression model by the following formula: risk score = (−0.3946 × *TP53* expression level) + (0.4447 × *CFL1* expression level) + (0.3412 × *UBA1* expression level). Based on the median value of the risk score, the patients were divided into the low-risk group and the high-risk group in the TCGA training cohort (Figure 7A). Meanwhile, the mortality of HCC patients increased with the prognostic risk score (Figures 7B,C) presented the heatmap of three prognostic genes, testifying the expression level of *CFL1* and *UBA1* were upregulated while the expression level of *TP53* was downregulated. In contrast to the low-risk patients, the high-risk group had poorer OS, revealed by KM survival curves (Figure 7D). The predictive accuracy of the three prognostic genes was further evaluated by the tROC in this cohort, with 3-year, and 5-year AUCs of 0.783 (C-index = 0.632–0.89), and 0.74 (C-index = 0.584–0.863), respectively, and showed larger AUC values compared with each gene above, which mean that the multi-gene model had better prediction ability in 3-year and 5-year OS (Figure 7E).

### Validation of the Prognostic Model in External Validation Patient Cohorts

**FIGURE 8:**
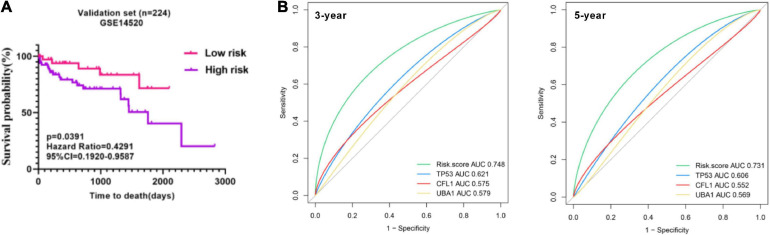
Presentation of the three-gene signature in the validation cohort. **(A)** Kaplan–Meier plots were applied to visualize the OS probabilities for the high-risk versus low-risk patients in the validation set (GSE14520). **(B)** Time-dependent ROC analysis of the three-gene signature in the validation set. OS, overall survival; ROC, receiver operating characteristic.

To prove the predictive value of the three-gene prognostic model, we adopted the GSE14520 as the external validation set (*n* = 224) to evaluate the results from the training set. In line with the results in the training set, the KM curves of the validation set showed that the high-risk group had worse prognosis than the low-risk group (*p* = 0.0391) (Figure 8A). tROC analysis showed that AUC for 3-year and 5-year OS of the external validation set were 0.748 (C-index = 0.644–0.838) and 0.731 (C-index = 0.673–0.831), respectively (Figure 8B), which also revealed more accurate AUC values in comparison with single gene. Collectedly, the three-signature prognostic model performed well in OS prediction for HBV carriers with HCC development.

### Development and Validation of a Survival-Predicting Nomogram

**FIGURE 9:**
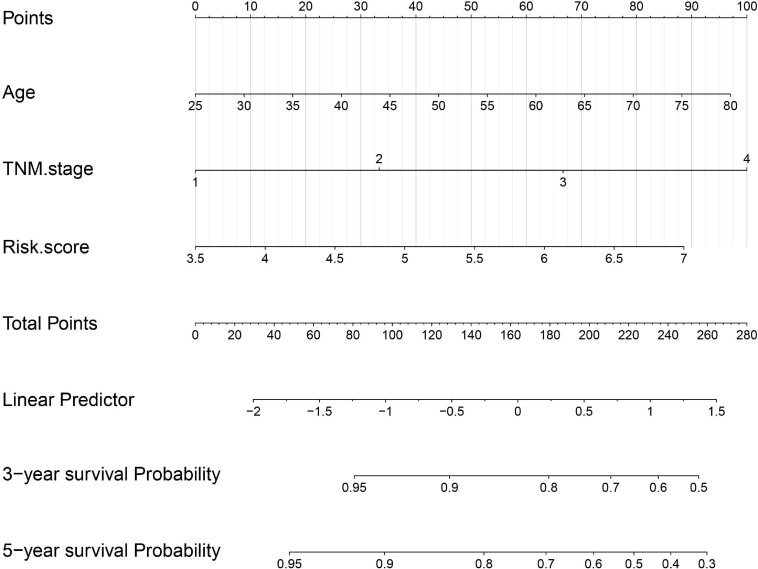
Nomogram construction integrated with risk score, age at diagnosis, and TNM stage to estimate the 3-year and 5-year survival probability for HBV carriers with HCC development.

**FIGURE 10:**
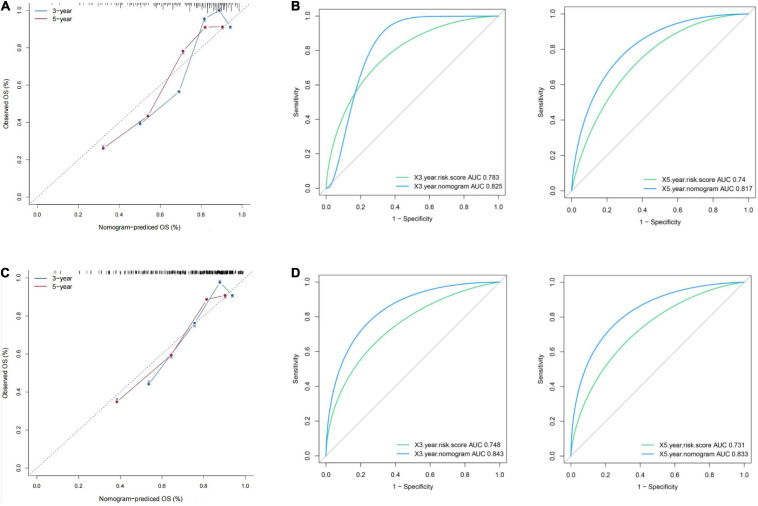
Presentation of nomogram to predict survival probability and comparison of the predictive power between nomogram and risk score. Calibration plots and time-dependent ROC curves of the nomogram for predicting the probability of OS at 3 years and 5 years in the training set **(A,B)** and the validation set **(C,D)**. OS, overall survival; ROC, receiver operating characteristic.

The risk score with other clinicopathological features (i.e., Gender, Age, Creatinine, Child-Pugh score, TNM stage, Ishak fibrosis score, Fetoprotein, Albumin, Bilirubin, Histologic grade, Platelet) was analyzed by the multivariate Cox proportional hazards regression method (Table 3). To establish a quantitative approach for OS prediction, a nomogram model was established in the TCGA training cohort. A point was calculated for each factor, and the total points of all factors were then obtained for the estimation of OS rates at 3 and 5 years (Figure 9). In addition, the calibration plot graphically showed a good agreement on the probability of 3-year and 5-year mortality between the prediction by nomogram and actual observation (Figure 10A). Consequently, the AUC value of 3-year and 5-year OS of nomogram was higher than that of risk score, which suggested that the three-gene prognostic nomogram may be an enhanced predictive capability after integrating the risk score with the age at diagnosis, TNM stage into a nomogram to predict OS (Figure 10B). To testify the predictive value of nomogram combined with risk score, age at diagnosis, and TNM stage, the validation set (*n* = 224) was assessed to verify the results above. There was also a good calibration curve for the mortality estimation in the validation cohort, consistent with that in the training cohort (Figure 10C). tROC curves of the risk score and three-gene based nomogram were compared with each other. Results showed that the nomogram had better 3-year or 5-year OS prediction than risk score in both the validation set and the training cohort (Figure 10D).

**TABLE 3:** Multivariate analysis of the overall survival in training cohort.

**Variable**	**HR**	**95%CI**	***p***
Risk score	2.000	1.083–3.693	**0.027**
Gender	1.016	0.982–1.051	0.360
Age at diagnosis	2.591	1.232–5.447	**0.012**
Creatinine	0.517	0.194–1.374	0.186
Child pugh classification grade	0.596	0.332–1.070	0.083
TNM stage	2.730	1.091–6.828	**0.032**
Fibrosis ishak score	1.994	0.861–4.620	0.107
Fetoprotein	0.708	0.345–1.451	0.345
Albumin	1.000	0.999–1.001	0.506
Bilirubin	1.226	0.754–1.994	0.412
Histologic grade	4877.72	0.000–526176	0.797
Platelet	0.483	0.226–2.370	0.059

*Bold indicates significant values (<0.05).*

## Discussion

For the most part, HBV infection gives rise to the development, invasion and metastasis of HCC, which is considered as a prevalent malignant liver disease (Study et al., 1997). At present, chronic HBV is one of the primary factors of HCC progression (Wood, 2011). Hence, HCC progression can be deemed as an end-stage consequence of chronic HBV infection (Ding et al., 2009). Thus, selection of early-phase prognostic signatures for HBV carriers are essential to allow effective treatments and prevent HCC from developing. The present study particularly concentrated on HBV–infected populations, which was disparate and innovative from the previous studies based on HCC patients.

In this study, dataset GSE83148 was utilized for WGCNA analysis to seek the key module, which was significantly enriched in multiple pathways, including arginine and proline metabolism, wnt signaling pathway, endometrial cancer, hepatitis B, and regulation of actin cytoskeleton. According to the previous studies, the pathways were closely related to the HBV infection (Jiang et al., 2014; Daud et al., 2017; Atteya et al., 2020). Through the univariate Cox regression analysis, 16 HBV-positive genes were related with HCC prognosis. Furthermore, three intersected genes (*TP53*, *CFL1*, and *UBA1*) were singled out with LASSO and SVM-RFE algorithms together. In order to certify the effectiveness and accuracy of the three HBV-associated genes during hepatocarcinogenesis, another microarray GSE114783 was utilized to explore the expression variations from healthy individuals, HBV carriers, chronic hepatitis B, liver cirrhosis, finally to liver cancer, these results demonstrated that the three genes (*TP53*, *CFL1*, *UBA1*) were aberrantly expressed during HCC development.

Our study revealed that the three genes (*TP53*, *CFL1*, *UBA1*) were all significantly related to the OS of HCC patients, which were in accord with the previous studies (Schulze et al., 2015; Shan et al., 2020; Zhang et al., 2021). Although all of the three signatures were not included in the previous signature panels, it was attributed to the former study that was focused populations on HCC patients rather than the HBV carriers. *TP53*, which can suppress the activity of cyclin-CDK, is closely connected with the accumulation of genetic changes. Besides, its proteins can conduct as tumor suppressors to control cell cycle in HBV-related HCC. *TP53* mutation is the most frequent mutation in HCC, which affects the progression and prognosis of HCC (J Bénard et al., 2010; Olivier et al., 2010). For example, in a meta-analysis about prognostic significance of *TP53* expression for HCC patients, it indicates that tumor p53 alterations are significantly associated with poor outcomes in HCC patients (Liu et al., 2012). Cofilin (*CFL1*) is regulated by multiple factors, such as phosphorylation, pH, binding of phosphoinositides, and subcellular compartmentalization, which can mediate apoptosis in response to oxidative stress (Ostrowska and Moraczewska, 2017). More importantly, *CFL1* is specifically accumulated in HBV-associated HCC (HBV-HCC) liver samples, which expression level is positively correlated with the severity of HBV-related liver disease. These results provide evidence that *CFL1* might be served as a potential biomarker for prognosis and diagnosis of HBV-HCCs (Xu et al., 2016). Ubiquitin-like modifier activating enzyme 1 (*UBA1*), the E1 ubiquitin-activating enzyme, sits at the apex of the ubiquitin cascade and represents an important regulator of cellular protein homeostasis (Kumbhar et al., 2018). The early findings had demonstrated that *UBA1* participated in the development of HCC by modulating Huh7 phenotypes and ferroptosis via the Nrf2 signal transduction pathway and might be a promising diagnostic and prognostic indicator for HCC (Shan et al., 2020). Thus, all of the above information suggested that they may play a coordinated role in HCC progression and were worthy of further investigation.

In our study, a risk score model based on the candidate hub genes’ expression levels and regression coefficients in the training cohort was established to predict prognosis for HBV carriers with HCC development, then which was validated in the external cohort. The patients in high-risk group showed significantly poorer prognosis than the patients in the low-risk group. And tROC curve analysis indicated that the three-signature panel could accurately predict the prognosis for HBV-related HCC patients, with the AUC of 0.783 and 0.74 in 3-year and 5-year OS for the training set, respectively, which was additionally sustained in the validation set. Furthermore, an enhanced predictive capability was detected after integrating the risk score with TNM stage and age at diagnosis into a nomogram model. Thus, the outcomes signified that the nomogram model could be used to stratify HCC patients with HBV infection into high-risk and low-risk group to help clinicians choose wiser clinical decisions.

Currently, there were several biomarkers or risk model for targeting HCC patients, which had achieved good results in clinical trial. While in our study, HBV carriers became the focus of our attention for the reason that patients with HBV–associated HCC had notably higher rates of metastasis and recurrence compared with those without HBV infection. Despite the reasonable generalizability and versatility of the risk model in OS prediction, several limitations in our study should be acknowledged. Firstly, a relatively small number of HBV-positive individuals were included to screen out HBV-associated genes to establish the risk model. Secondly, the clinicopathological parameters were not taken into account to classify the HCC patients into diverse risk groups to compare the difference of OS.

## Conclusion

In our current study, we identified a risk model based on three prognostic signatures (*TP53*, *CFL1*, and *UBA1*) from publicly available data and further constructed a three-gene based prognostic nomogram which contained other clinical parameters (TNM stage and age at diagnosis) to predict 3-year and 5-year OS of HBV carriers with HCC development, which may be more effective in guiding the clinical decision-making of personalized treatment.

## Data Availability Statement

The datasets presented in this study can be found in online repositories. The names of the repository/repositories and accession number(s) can be found in the article/supplementary material.

## Author Contributions

YH conceived of the study idea. Y-LC and Y-QY collected the data to be analyzed. YH and W-LT performed the data analysis and produced the results. YH, W-LT, and L-PM wrote and revised the manuscript. All authors discussed the results and contributed to the final manuscript.

## Conflict of Interest

The authors declare that the research was conducted in the absence of any commercial or financial relationships that could be construed as a potential conflict of interest.

## Publisher’s Note

All claims expressed in this article are solely those of the authors and do not necessarily represent those of their affiliated organizations, or those of the publisher, the editors and the reviewers. Any product that may be evaluated in this article, or claim that may be made by its manufacturer, is not guaranteed or endorsed by the publisher.

## Footnotes

1
http://www.ncbi.nlm.nih.gov/geo

2
https://portal.gdc.cancer.gov/

3
https://cran.r-project.org/web/packages/WGCNA/index.html

4
http://www.bioconductor.org/packages/release/bioc/html/clusterProfiler.html

5
https://string-db.org/

6
https://cran.r-project.org/web/packages/glmnet/index.html

7
https://cran.r-project.org/web/packages/e1071/index.html

8
https://www.proteinatlas.org/

9
http://kmplot.com/analysis

10
https://cran.r-project.org/web/packages/ggpubr/index.html

11
https://cran.r-project.org/web/packages/pROC/index.html

12
https://cran.r-project.org/web/packages/rms/index.html
